# Cognitive Screening in Brain Tumors: Short but Sensitive Enough?

**DOI:** 10.3389/fonc.2015.00060

**Published:** 2015-03-11

**Authors:** Gail A. Robinson, Vivien Biggs, David G. Walker

**Affiliations:** ^1^Neuropsychology Research Unit, School of Psychology, The University of Queensland, Brisbane, QLD, Australia; ^2^BrizBrain and Spine, The Wesley Hospital, Brisbane, QLD, Australia

**Keywords:** neurocognitive deficits, brain tumor, cognitive screening, neuropsychology, brief cognitive assessment, MoCA

## Abstract

Cognitive deficits in brain tumors are generally thought to be relatively mild and non-specific, although recent evidence challenges this notion. One possibility is that cognitive screening tools are being used to assess cognitive functions but their sensitivity to detect cognitive impairment may be limited. For improved sensitivity to recognize mild and/or focal cognitive deficits in brain tumors, neuropsychological evaluation tailored to detect specific impairments has been thought crucial. This study investigates the sensitivity of a cognitive screening tool, the Montreal Cognitive Assessment (MoCA), compared to a brief but tailored cognitive assessment (CA) for identifying cognitive deficits in an unselected primary brain tumor sample (i.e., low/high-grade gliomas, meningiomas). Performance is compared on broad measures of impairment: (a) number of patients impaired on the global screening measure or in any cognitive domain; and (b) number of cognitive domains impaired and specific analyses of MoCA-Intact and MoCA-Impaired patients on specific cognitive tests. The MoCA-Impaired group obtained lower naming and word fluency scores than the MoCA-Intact group, but otherwise performed comparably on cognitive tests. Overall, based on our results from patients with brain tumor, the MoCA has extremely poor sensitivity for detecting cognitive impairments and a brief but tailored CA is necessary. These findings will be discussed in relation to broader issues for clinical management and planning, as well as specific considerations for neuropsychological assessment of brain tumor patients.

## Introduction

Cognitive function is an independent prognostic factor in the survival of glioma patients (1, 2). For brain tumors, cognitive assessment (CA) can inform clinicians of areas to target for neurorehabilitation (3), monitor progress to facilitate decision making about further intervention (4), and if there has been a decline in cognitive function, address the question of whether the tumor has recurred or progressed (3). In addition, a CA is able to address the question of whether subtle alterations in cognitive function are significant or not, particularly when monitoring slow-growing low-grade gliomas (4). Assessment of cognitive status can be undertaken with a brief cognitive screen or by a longer formal neuropsychological evaluation. Cognitive screening is typically used in acute states, at bedside, hence the focus of our study is to identify whether a brief CA can be tolerated and completed in a relatively acute state (post-surgery but <3 months) and, if so, whether this yields better results in terms of detecting cognitive deficits.

Cognitive screening tools are popular but their sensitivity to cognitive impairment in general, and specifically for brain tumor patients, has been questioned (4). One reason may be that brain tumor-associated cognitive deficits have been thought to be relatively mild and non-specific (5), although this has recently been challenged (6). It is unsurprising that severity and specificity of cognitive deficits in brain tumor patients has been debated as prevalence rates vary from 29 to 91%. This variability may depend on several factors including time of assessment (pre- or post-surgery), tumor grade, treatments (radiation, chemotherapy), and lesion location (7). However, the main reason for this variability may be the method used to assess cognitive functions. For example, in one study, few patients with low-grade gliomas showed cognitive deterioration when screened with the mini-mental state examination (MMSE) (8), irrespective of radiation treatment (9). By contrast, Tucha and colleagues (10) investigated cognitive function with neuropsychological tests and reported that 91% of patients with frontal or temporal tumors were impaired in at least one cognitive domain. In this study, we aimed to investigate the most effective and efficient method of detection of cognitive impairments in the acute period following tumor resection by directly comparing a cognitive screening tool with a brief but domain-specific CA.

Cognitive screening tools have the advantage of brevity and simplicity of administration. The main question, however, is whether these tools are sensitive to detect abnormalities. In the last decade, the Montreal Cognitive Assessment (MoCA) (11) screening tool has been increasingly favored over the MMSE as it has been shown to have greater sensitivity for detecting cognitive dysfunction. This has been shown in patients with brain tumors (12) and brain metastases (13), as well as in other neurological conditions including stroke (14), sub-arachnoid hemorrhages (SAHs) (15), and silent cerebral infarcts (16). Bernstein et al. investigated the psychometric properties of the MoCA in three diverse brain pathologies and concluded that it was reliable in detecting cognitive dysfunction as well as having the benefit of not fatiguing the patient (17). However, regardless of which cognitive screening tool has the greatest sensitivity, the original purpose of these tools was to detect global or generalized decline rather than domain-specific cognitive deficits. Indeed, the need for domain-specific cognitive tests for the brain tumor population was recently highlighted by a study of glioma patients (6). In this study, a range of specific visuospatial deficits were identified in right parieto-temporal gliomas that were not present in patients with prefrontal tumors Thus, it remains uncertain whether cognitive screening tools are sensitive to identify mild and/or focal deficits in brain tumors (4, 12).

Neuropsychological evaluations are held to be the “gold standard” for assessment of cognitive functions in focal neurological disorders like stroke (15, 18). However, evaluations differ in test composition and can range from long and comprehensive, with a fixed test battery, to brief and flexible, with tests chosen to assess specific cognitive domains (19). One advantage of neuropsychological evaluation is the freedom to include tests that tap specific cognitive functions, depending on tumor location and presenting symptoms (4). On the other hand, the main criticism is the length of assessment that can range from brief (1–2 h) to lengthy (8 h). Length is a particular issue in brain tumor patients as physical and mental fatigue has specifically been identified as a concern (12, 20). In fact, Olsen and colleagues (12) found a selection bias in which patients were willing to complete a 4-h neuropsychological assessment. In particular, they identified that those who completed both the 4-h assessment and cognitive screening tests, tended to be younger with a higher level of education, they obtained a higher MoCA score and were on lower doses of medications. Thus, Olsen and colleagues, like Papagno et al. (4), concluded that a brief and well-tolerated CA is desirable, when diagnostic accuracy can be maintained.

Neuropsychological evaluation has been compared to cognitive screening tools. As noted above, Olsen and colleagues (12) compared neuropsychological assessment to both the MMSE and the MoCA. The MoCA showed greater sensitivity to cognitive dysfunction than the MMSE; however, the main conclusion was that inclusion of a 4-h neuropsychological assessment was a significant deterrent for participation. The MMSE and MoCA, compared to neuropsychological assessment and return to work status, have been investigated in patients following aneurysmal SAH (15). In their study, 42% of patients were impaired on the MoCA, compared to none on the MMSE, and the MoCA correlated with domain-specific cognitive tests while the MMSE showed no association with specific tests. In addition, two MoCA items were associated with return to work. The MoCA was concluded to be more sensitive than the MMSE in SAH; however, it was not clear that the MoCA had sufficient sensitivity when compared to the neuropsychological assessment (15). Recently, a large retrospective study of acute stroke has unequivocally demonstrated that the MoCA underestimated cognitive impairment, compared to a brief 1–2 h neuropsychological assessment (18).

The current study compared the MoCA cognitive screening tool with a brief 1–1.5 h neuropsychological evaluation in primary brain tumors. The neuropsychological evaluation comprised a CA and mood and behavioral assessments as this is thought important to fully characterize level of function and inform care plans (7). The aim was to ascertain whether the MoCA is sufficiently sensitive to detect cognitive impairment at an acute, post-resection time point or whether a brief but domain-specific CA is necessary.

## Materials and Methods

### Patients

Thirty-six patients with primary brain tumors (low- or high-grade gliomas, meningiomas) were recruited by the Brain Tumor Nurse Practitioner (VB) from BrizBrain and Spine, The Wesley Hospital, Brisbane, QLD, Australia. Ethical approval for the study was granted by the UnitingCare and The University of Queensland Human Research Ethics Committees. Informed and written consent was obtained from all patients. Inclusion criteria were (1) confirmation of brain tumor ascertained by MRI and (2) all patients underwent surgical resection prior to the investigation of cognitive functions. The cognitive screening tool was administered before the CA, which was completed in one testing session. The third (3) inclusion criterion was that the cognitive screening tool and CA were completed within the same week to minimize effects due to timing of cognitive screening or assessment. Thus, due to the latter, only 23 patients aged 18–69 years old were included. The mean time between surgical resection and neuropsychological evaluation was 2.1 months (SD = 3.1; see Table 1 for patient characteristics). We note that 2.1 months is sufficient time to allow for findings to be useful for neurorehabilitation (if available), planning for management of deficits for the patient and family/carers, and to address any questions related to returning to community roles at home or work.

**Table 1:** **MoCA, demographic, and behavioral scores (mean ± SD): all patients and MoCA sub-groups (MoCA-Intact and MoCA-Impaired)**.

	All (*N* = 23)	MoCA-Intact (*N* = 16)	MoCA-Impaired (*N* = 7)
MoCA score (/30)	26.52 ± 2.11	27.63 ± 1.15	24.00 ± 1.53***
Age (*M* ± SD)	48.39 ± 14.61	46.94 ± 15.81	51.71 ± 11.79
Gender (M:F)	16:7	12:4	4:3
Education	13.90 ± 2.98	14.33 ± 2.94	12.83 ± 3.06
Pre-morbid estimated intelligence (NART IQ)	104.13 ± 10.35	103.56 ± 10.24	105.43 ± 11.30
Chronicity (months post-surgery)	2.07 ± 3.11	2.54 ± 3.48	0.67 ± 0.41
Tumor type (WHO grade)
Meningioma	4	3	1
Oligodendroglioma (II)	5	4	1
Astrocytoma (II)	4	2	2
Oligodendroglioma (III)	1	1	0
Astrocytoma (III)	1	1	0
Glioblastoma multiforme (IV)	8	5	3
Tumor location (L/R)	12/11	8/8	4/3
Frontal (L/R)	7/3	4/2	3/1
Temporal (L/R)	2/3	1/2	1/1
Parietal (L/R)	0/2	0/2	0/0
>1 lobe (L/R)	2/3	2/2	0/1
HADS anxiety (/21)	5.94 ± 3.86	5.83 ± 4.17	6.20 ± 3.43
HADS depression (/21)	3.88 ± 2.62	3.08 ± 2.43	5.80 ± 2.17*
Apathy Evaluation Scale (/72)	49.20 ± 15.41	50.86 ± 16.17	45.33 ± 14.05

NART, National Adult Reading Test; L, left; R, right; HADS, Hospital Anxiety and Depression Scale. ***p < 0.001, *p < 0.05, MoCA-Intact compared to MoCA-Impaired patients.

### Cognitive screening

The MoCA (11) was used as the screening tool. Although it was developed as a brief measure of global cognitive function, it contains items that measure these cognitive domains: visuospatial/executive function; naming; memory; language; abstraction; and attention. Specifically, the MoCA is scored out of 30 points comprising these items: brief trail making, cube copy, and clock drawing (visuospatial/executive domain = 5 pts); animals to name (naming domain = 3 pts); five words to recall (memory domain = 5 pts); three brief attention tasks (attention domain = 6 pts); sentence repetition and word fluency (language domain = 3 pts); similarities (abstraction domain = 2 pts); time/place questions (orientation domain = 6 pts). A normal score is 26 or above.

### Neuropsychological evaluation

#### Cognitive assessment

A brief but tailored CA was administered that was completed in 1–1.5 h, depending on individual patient’s level of fatigue and ability. The CA was devised based on neuropsychological assessment principles and assessment of standard cognitive domains, detailed in Cipolotti and Warrington (21). The cognitive tests were specifically chosen based on Robinson’s recent lesion studies of brain tumor and stroke patients with focal frontal and non-frontal lesions [e.g., Ref. (22, 23)]. A similar approach was adopted by Papagno and colleagues (4) in their recent study of low-grade gliomas. Thus, estimated pre-morbid level of intelligence was ascertained in a standard manner by administering the National Adult Reading Test (NART) (24). To ascertain current level of cognitive function, the CA comprised standard published neuropsychological tests that focused on the following domain-specific areas of cognition: (1) *Abstract reasoning*: non-verbal – Raven’s advanced progressive matrices (25), verbal – Proverb Interpretation Test (26, 27); (2) *attention* – Digit Span subtest from the Wechsler Adult Intelligence Scale-III (28), Elevator Counting with Distraction from the Test of Everyday Attention (29); (3) *verbal and visual memory* – Recognition Memory Tests, Words, and Topography (30, 31); (4) *visual perception* – Incomplete Letters Test from the Visual Object and Space Perception Battery (32); (5) *language* – Graded Naming Test (33), Word Comprehension – Synonyms Test (34); and (6) *executive functions* – phonemic word fluency (35), Hayling Sentence Completion Test (36).

#### Mood and behavior assessment

As part of the neuropsychological evaluation, level of self-reported anxiety, depression, and apathy were assessed using the Hospital Anxiety and Depression Scale (HADS) (37) and the Apathy Evaluation Scale (AES) (38). A score on the HADS of 7 or below is in the normal range with a score at or above 11 indicating significant levels of anxiety or depression. The AES results in scores between 18 and 72, with higher scores indicating increased apathy and a score of 41 suggested as the cut-off.

#### Analyses

The MoCA and domain-specific cognitive tests were administered and scored in the standard and published manner. Patients were classified as cognitively intact on the MoCA if they obtained a score of ≥26 or impaired if they scored <26 (11). For each individual cognitive test, patients were classified as cognitively impaired if they scored <5th percentile (i.e., 5% cut-off), with an intact performance ≥5% cut-off [for similar methodology, see Ref. (18, 39)]. For the Proverb Interpretation Test of verbal abstraction, an impaired performance was a score of <5/8 [for scoring details, see Ref. (24)].

Performance was analyzed in several ways. First, we calculated a broad measure of impairment for both the MoCA and the CA. For the MoCA, the number of patients impaired is reported. For the CA, we calculated the number of patients impaired on any test and also the number of cognitive domains each patient was impaired in (i.e., 0–6 cognitive domains). Second, based on the method adopted by Chan and colleagues for stroke patients (18), we conducted two specific analyses: (1) MoCA-Intact patients were investigated for impairment in each cognitive domain assessed by the CA; and (2) Patients who scored the maximum points in each of the MoCA-specified cognitive domains, irrespective of the overall MoCA score, were analyzed in terms of discrepancy between this and performance on the domain relevant CA test. We also analyzed whether the MoCA-Impaired patients were impaired in at least one cognitive domain.

## Results

For the first broad measure, we found that 30.4% (7/23) of our patients were impaired on the MoCA as they scored <26. A summary of the MoCA, demographic, and mood and behavior scores for the whole group, and the MoCA-Intact and MoCA-Impaired sub-groups, are contained in Table 1. As expected, the MoCA score for the impaired group was significantly lower than the intact group, *t*(21) = 6.31, *p* < 0.001. Apart from slightly higher self-reported symptoms of depression by the MoCA-Impaired group compared to the MoCA-Intact group, *t*(15) = 2.16, *p* < 0.05, the two groups were well matched for age, gender, education, pre-morbid intelligence, and chronicity (time since surgery; all *p* > 0.05). Similarly, there was no difference between these two groups in self-reported anxiety or apathy. With regard to symptoms of depression, we note that the mean of both groups is in the “normal” range and not indicative of clinical or sub-clinical depression. If we examine individual scores, one patient in each group (MoCA-Intact and -Impaired) was in the abnormal range. For anxiety, abnormal scores were obtained by three patients in each group (MoCA-Intact and -Impaired). Finally, both groups reported mildly elevated levels of apathy with a number of patients in both groups above the suggested cut-off (11 in the MoCA-Intact and 4 in the MoCA-Impaired group), which may reflect the acute post-resection stage of assessment.

For the CA broad measure, 69.6% (16/23) of the patients were impaired on at least one domain-specific cognitive test. The means and SDs for the whole group, and the MoCA-Intact and MoCA-Impaired sub-groups, are reported in Table 2. Overall, there was no difference between sub-groups in performance on 9 of the 11 cognitive tests (i.e., *p* > 0.05), which supports specific patterns of cognitive deficits rather than a generally lower performance of the MoCA-Impaired patients. By contrast, the MoCA-Impaired group performed significantly poorer on the Graded Naming Test of language, *t*(21) = 2.567, *p* < 0.05, and the phonemic word fluency test that is sensitive to executive dysfunction, *t*(21) = 2.363, *p* < 0.05. The number of cognitive domains each patient was impaired in was as follows: 4/16 impaired in one domain; 4/16 impaired in two domains; 6/16 impaired in three domains; and 2/16 impaired in four domains. Thus, 75.0% of the impaired patients were impaired on tests in *at least* two cognitive domains.

**Table 2:** **Domain-specific Cognitive Test Scores (mean ± SD): all participants, MoCA sub-groups (MoCA-Intact and MoCA-Impaired), and comparison statistic between MoCA sub-groups**.

Cognitive domain/test	All (*N* = 23)	MoCA-Intact (*N* = 16)	MoCA-Impaired (*N* = 7)	*p* Value
**Abstract reasoning**
Advanced progressive matrices (/12)	7.09 ± 2.15	7.31 ± 2.27	6.57 ± 1.90	*p* = 0.460
Proverb Interpretation Test (/8)	4.47 ± 1.28	4.67 ± 1.16	4.00 ± 1.58	*p* = 0.344
**Memory**
RMT words (/50)	45.91 ± 4.51	46.19 ± 4.52	45.29 ± 4.79	*p* = 0.670
RMT topography (/30)	22.57 ± 6.33	23.40 ± 6.24	20.50 ± 6.63	*p* = 0.356
**Attention**
Digit span total	17.00 ± 5.14	17.00 ± 4.45	17.00 ± 6.99	*p* = 1.00
Elevator counting + distraction (/10)	5.88 ± 3.38	6.20 ± 3.33	5.33 ± 3.72	*p* = 0.095
**Language**
Graded Naming Test (/30)	18.22 ± 3.46	19.31 ± 2.82	15.71 ± 3.68*	***p* = 0.018**
Synonyms (/50)	39.85 ± 3.66	40.36 ± 3.75	38.67 ± 3.45	*p* = 0.358
**Visual perception**
Incomplete letters (/20)	19.50 ± 0.67	19.56 ± 0.73	19.33 ± 0.52	*p* = 0.490
**Executive function**
Phonemic word fluency (FAS)	34.25 ± 13.23	38.36 ± 11.91	24.67 ± 11.78*	***p* = 0.030**
Hayling Test Overall Scaled Score (1–10, 6 = Average)	3.52 ± 2.47	3.63 ± 2.36	3.29 ± 2.87	*p* = 0.769

Bold represents a significant finding. *p < 0.05.

**Figure 1:**
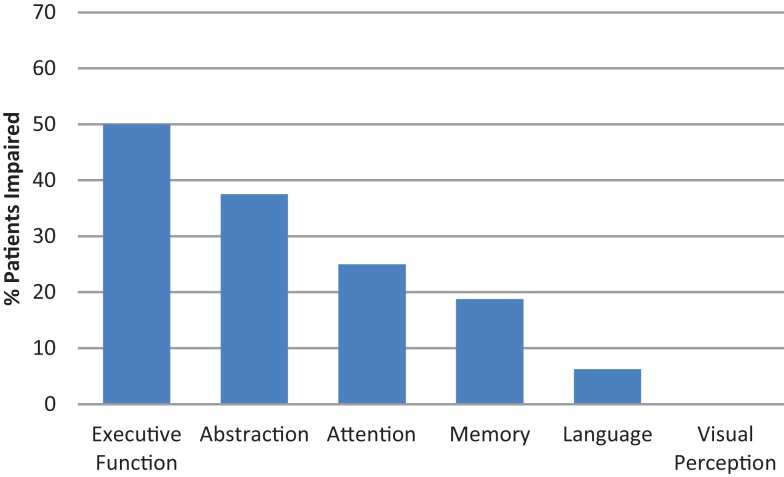
**Cognitive assessment: MoCA-Intact participants impaired in domain-specific cognitive tests**.

For the specific measures based on Chan et al. (18), first we investigated the 16 MoCA-Intact patients for impairment in each cognitive domain assessed by the CA. Of these patients, 56.3% were impaired in at least one of the six cognitive domains. The percentage of MoCA-Intact patients impaired on domain-specific cognitive tests is shown in Figure 1. The main cognitive domains impaired for MoCA-Intact patients were abilities related to higher level executive functions, including abstract reasoning, followed by attention and memory. By contrast, language was only impaired in <10% and no patient was impaired on the test of visual perception.

For the second specific measure, we examined patients who scored the maximum points in each of the MoCA-specified cognitive domains, irrespective of the overall MoCA score. Based on Chan et al. (18), we analyzed the discrepancy between this and performance on the domain relevant CA test. The number of patients who scored full marks on each MoCA-specified domain, were impaired on the relevant CA test and the negative predictive values are reported in Table 3.

**Table 3:** **Cognitive assessment performance and negative predictive value for MoCA-specified domains**.

MoCA-specified domain	No. patients scoring full marks on MoCA	No. patients (%) impaired on CA	Negative predictive value (NPV)
Visuospatial/executive	13	5 (38%)	0.62
Naming	21	3 (14%)	0.86
Memory	4	1 (25%)	0.75
Attention	13	4 (31%)	0.69
Language	7	3 (43%)	0.57
Abstraction	22	9 (41%)	0.59

For the MoCA-Impaired patients, 100% were impaired in at least one cognitive domain on the CA. Thus, when a patient obtains an impaired score on the MoCA this fully predicts significant impairment in at least one domain on CA. By contrast, the implications for cognitive function is less certain when a “normal” MoCA score is obtained as the MoCA showed very poor negative predictive value (0.44). Further, sensitivity for detecting cognitive impairment is extremely poor (0.44) in our primary brain tumor sample.

## Discussion

In our unselected primary brain tumor sample, only 30.4% were impaired on the MoCA cognitive screening tool. By contrast, for the CA, 69.6% of patients were impaired on at least one domain-specific cognitive test and, of these, 75% were impaired in at least two cognitive domains. If we examine the MoCA-Intact patients, more than half (56.3%) were impaired in at least one of the six cognitive domains. Specifically, 50% of the MoCA-Intact patients were impaired on tests of executive function, including abstraction, and a quarter of these patients were impaired in the domains of attention and memory. The level of sensitivity of 0.44 for the MoCA in our patients was far lower than for other neurological disorders. For example, the sensitivity of the MoCA in an acute stroke population was 0.82 (18) and, notably, assessments were completed at comparable times post-stroke or tumor resection. However, we note that the MoCA has been found useful in patients with brain metastases (13) and it is reported to be adequate for the detection of mild cognitive impairment in neurodegenerative disorders such as Alzheimer’s and Parkinson’s disease [e.g., Ref. (40)]. Nevertheless, the sensitivity of 0.44 of the MoCA for our primary brain tumor population is *extremely poor*.

In light of this low detection rate of cognitive abnormalities, it is noteworthy that the mean MoCA score of 26.5 for our tumor patients is relatively high and indicative of mild global cognitive impairment. This was also the case for our mood and behavioral measures of anxiety, depression, and apathy. More specifically, the “MoCA-Intact” group obtained a score almost identical to the normal controls reported by Nasreddine et al. (11) while the mean score of 24 for the “MoCA-Impaired” group falls toward the top of the “mild cognitive impairment” group. The overall “mild” level of impairment on the MoCA in our sample differs from the lower MoCA mean score of 22 in patients with brain metastases (13). In fact, Olsen et al. suggested that the MoCA score may be helpful in this population as patients with low MoCA scores may be less likely to benefit from palliative whole-brain radiotherapy while patients with high MoCA scores may tolerate more intensive interventions (13). Thus, for prognostic and treatment purposes in brain metastases, the MoCA may be useful. However, our results at a global level support the notion that primary brain tumor-associated cognitive deficits are indeed mild and/or focal and are hard to detect using global screening tools like the MoCA.

For the 69.6% of patients impaired in at least one cognitive domain on the CA, executive functions and abstract reasoning were the most common domains impaired by far. In fact, 87.5% of patients were impaired in these two domains and the remaining two patients presented with a selective nominal aphasia. This is followed by attention (43.8% impaired) and memory (37.5% impaired). These cognitive domains being the most often impaired is consistent with the findings of Tucha et al. (10) for frontal and temporal tumor patients. Interestingly, of the two executive function tests, phonemic word fluency and the Hayling Test, 52.2% of *all* patients were impaired on just one test, the Hayling Test, which suggests that test choice is critical. With regard to memory, the MoCA does not assess visual memory and 21.7% of our patients were impaired on our specific visual memory test. By contrast, the intact performance of all our patients on our test of visual perception does not reflect the finding of Shallice and colleagues (6) of visuospatial deficits in right posterior tumor patients. There are two possibilities for the apparent disparity. One, our specific test of visual perception is not sensitive to mild deficits. Two, our seven patients with right posterior tumors are remarkably intact. Upon examination of individual patients, one right temporal MoCA-Impaired and three right posterior MoCA-Intact patients lost points on the MoCA-specified visuospatial items. In addition, one of the MoCA-Intact patients presented with a highly selective apperceptive amusia in the context of an otherwise intact cognitive profile (41). This latter case, in addition to the two patients with a selective nominal aphasia, highlight the potential for any cognitive deficit to be specific and focal in brain tumor patients, thus, necessitating freedom in test choice based on symptoms and/or tumor location.

Notably, patients who performed well on MoCA-specified domains were not always intact on the specific cognitive test, similar to Chan et al.’s findings in acute stroke patients. This is particularly so for the abstraction and executive/visuospatial MoCA-specified domains that are assessed by one item each, both clearly insensitive to our patients’ deficits. By contrast, the two MoCA-specified domains that most closely resembled the CA impairments were language and memory. In terms of language, only 30.4% of patients scored full marks on the MoCA-specified items that comprised a sentence repetition item (>10 words in length) and a phonemic word fluency task. Of these two items, phonemic word fluency was one of two standard cognitive tests that MoCA-Impaired patients performed significantly poorer than MoCA-Intact patients and it can be classed a test of executive function. If we examine naming ability, almost all patients obtained full marks for the MoCA naming items, although 17.4% of all patients were impaired on the standard Graded Naming Test. Very few patients obtained full marks on the MoCA-specified memory items although, as noted above, a main limitation of the MoCA is that visual memory is not assessed.

The inclusion of all types of brain tumors in our study could be argued to limit our findings. This is unlikely for two reasons. First, in our study, patients with both meningiomas and gliomas (high/low-grade) were in the MoCA-impaired group (see Table 1). Secondly, in a recent study specifically investigating the effect of etiology on cognitive performance in patients with focal frontal lesion, once age and pre-morbid intelligence were accounted for, there were no significant differences between patients of different etiologies (stroke, meningioma, high/low-grade gliomas (42). One caveat, however, is the practical implications of treatments for different brain tumor types. For example, the timing of a brief CA in patients with higher grade gliomas who proceed to receive initial radiation or chemotherapy at 2–6 weeks post-resection, followed by a gap with no treatment and then adjuvant chemotherapy (43), needs to be considered in specific contexts. If a neuropsychologist is attached to an acute neurosurgical ward, then assessment prior to treatment can be included in routine planned care. If this is unavailable, then an optimal time would be in the gap between treatments, which would be approximately 8–10 weeks post-resection. Findings from a brief CA at either of these time points will be useful in further management, informing specific cognitive strategies/interventions and for the patient to understand changes in thinking related to their tumor.

In summary, the MoCA has extremely poor sensitivity to cognitive impairment in our primary brain tumor sample, which means that if a “normal” MoCA score is obtained, a CA is necessary. Even if a patient is impaired on the MoCA, the severity may be underestimated and some areas of cognition are not assessed. In fact, only one MoCA-specified domain showed even remotely similar detection levels as a brief CA. A full discussion of other brief cognitive screening tools (e.g., ACE-III; CogMed) is beyond the scope of this preliminary study although we can speculate that similar issues would be revealed. Thus, despite the limitations of our small sample size, we strongly demonstrate that a brief and tailored CA lasting only 1–1.5 h is necessary and possible for the detection of cognitive impairments in primary brain tumor patients in the acute phase post-surgery. This is not only important for prognosis and monitoring, but it is crucial for neurorehabilitation and interventions (1, 2, 4). Moreover, mental deterioration, or fear of this, was rated as one of the highest concerns of patients and carers, contributing to quality of life (20). Our study suggests that the critical cognitive domains to assess are executive functions (initiation, suppression, abstraction), attention, memory (verbal and visual), and language (naming and verbal fluency). Finally, we highly recommend adopting the neuropsychological principle of tailoring an assessment based on lesion location and presenting symptoms.

## Conflict of Interest Statement

The authors declare that the research was conducted in the absence of any commercial or financial relationships that could be construed as a potential conflict of interest.
